# Hydrogen Sulfide Is a Novel Protector of the Retinal Glycocalyx and Endothelial Permeability Barrier

**DOI:** 10.3389/fcell.2021.724905

**Published:** 2021-09-07

**Authors:** Claire L. Allen, Katarzyna Wolanska, Naseeb K. Malhi, Andrew V. Benest, Mark E. Wood, Winfried Amoaku, Roberta Torregrossa, Matthew Whiteman, David O. Bates, Jacqueline L. Whatmore

**Affiliations:** ^1^Cancer Biology, Division of Cancer and Stem Cells, School of Medicine, Biodiscovery Institute, University of Nottingham, Nottingham, United Kingdom; ^2^The Institute of Biomedical and Clinical Science, University of Exeter Medical School, St. Luke’s Campus, University of Exeter, Exeter, United Kingdom; ^3^Biosciences, College of Life and Environmental Science, University of Exeter, Exeter, United Kingdom; ^4^Academic Ophthalmology, Division of Clinical Neuroscience, School of Medicine, University of Nottingham, Nottingham, United Kingdom

**Keywords:** glycocalyx, retinal permeability, diabetes, hydrogen sulfide, inflammation, mitochondria, slow-release hydrogen sulfide donors

## Abstract

Significantly reduced levels of the anti-inflammatory gaseous transmitter hydrogen sulfide (H_2_S) are observed in diabetic patients and correlate with microvascular dysfunction. H_2_S may protect the microvasculature by preventing loss of the endothelial glycocalyx. We tested the hypothesis that H_2_S could prevent or treat retinal microvascular endothelial dysfunction in diabetes. Bovine retinal endothelial cells (BRECs) were exposed to normal (NG, 5.5 mmol/L) or high glucose (HG, 25 mmol/L) ± the slow-release H_2_S donor NaGYY4137 *in vitro*. Glycocalyx coverage (stained with WGA-FITC) and calcein-labeled monocyte adherence were measured. *In vivo*, fundus fluorescein angiography (FFA) was performed in normal and streptozotocin-induced (STZ) diabetic rats. Animals received intraocular injection of NaGYY4137 (1 μM) or the mitochondrial-targeted H_2_S donor AP39 (100 nM) simultaneously with STZ (prevention) or on day 6 after STZ (treatment), and the ratio of interstitial to vascular fluorescence was used to estimate apparent permeability. NaGYY4137 prevented HG-induced loss of BREC glycocalyx, increased monocyte binding to BRECs (*p* ≤ 0.001), and increased overall glycocalyx coverage (*p* ≤ 0.001). In rats, the STZ-induced increase in apparent retinal vascular permeability (*p* ≤ 0.01) was significantly prevented by pre-treatment with NaGYY4137 and AP39 (*p* < 0.05) and stabilized by their post-STZ administration. NaGYY4137 also reduced the number of acellular capillaries (collagen IV + /IB4-) in the diabetic retina in both groups (*p* ≤ 0.05). We conclude that NaGYY4137 and AP39 protected the retinal glycocalyx and endothelial permeability barrier from diabetes-associated loss of integrity and reduced the progression of diabetic retinopathy (DR). Hydrogen sulfide donors that target the glycocalyx may therefore be a therapeutic candidate for DR.

## Introduction

Loss of integrity of the vascular permeability barrier is associated with a range of pathological conditions. In particular, increased permeability is observed in diabetes, and in hyperglycemic conditions (Gillies et al., 1997; Brownlee, 2001; Allen and Bayraktutan, 2009; Saker et al., 2014) where it is associated with the pathogenesis of life-altering diabetic microvascular complications such as retinopathy (Sander et al., 2007). The vascular permeability barrier is formed of endothelial cells (ECs), their underlying basement membrane, and the endothelial glycocalyx. The latter is now recognized as a key regulator of permeability as demonstrated by a range of studies showing that selective loss of specific glycocalyx components is associated with altered permeability properties in various vascular beds (Jeansson and Haraldsson, 2003; Singh et al., 2007; Landsverk et al., 2012; Betteridge et al., 2017; Onions et al., 2019).

A role for endothelial glycocalyx damage in diabetes-associated vascular changes has been described. Glycocalyx reduction is associated with increased vascular permeability and increased leucocyte and platelet adhesion in acute hyperglycemia and diabetes (Nieuwdorp et al., 2006a,b; Broekhuizen et al., 2010; Kumase et al., 2010), which were reversed by glycocalyx restoration experimentally in animals (Henry and Duling, 1999; Constantinescu et al., 2003). Strategies to reverse or reduce glycocalyx damage in diabetes have the potential to rescue some of the potentially damaging vascular changes that underlie the development of vascular complications such as retinopathy.

We hypothesized that exogenous application of hydrogen sulfide (H_2_S) may protect the glycocalyx in diabetes, reversing or preventing the retinal vascular leakage observed in diabetic retinopathy (DR). Hydrogen sulfide has recently been identified as a seemingly ubiquitous “gaseous mediator” in mammals and humans where it is synthesized by multiple cell types by at least four distinct enzyme systems utilizing endogenous sulfur-containing amino acids. These systems include the cytosolic cystathionase (CSE), cystathionine-β-synthase (CBS), mitochondrial cysteine aminotransferase/3-mercaptopyruvate sulfurtransferase (CAT/3MST), and D-amino acid oxidase/3- mercaptopyruvate sulfurtransferase (DAO/3MST) (Whiteman et al., 2011; Szabo, 2012; Wang, 2012; Szabo et al., 2014). The first three, at least have been demonstrated in ECs (Wang, 2012; Tao et al., 2013; Mitidieri et al., 2020). Experimentally, manipulation of H_2_S level has been achieved by pharmacological inhibition or genetic removal of enzyme systems for H_2_S and the use of H_2_S delivery molecules, albeit generally limited to the use of simple inorganic sulfide salts [e.g., sodium hydrosulfide (NaSH) or disodium sulfide (Na_2_S)]. These studies have revealed that H_2_S and/or physiologically derived species exert a wide variety of effects on different organ systems including regulation of vascular tone (Yang et al., 2008; Kanagy et al., 2017; Szabo, 2017), inflammation (Whiteman and Winyard, 2011), aging and health span (Ng et al., 2018; Zivanovic et al., 2019), and more recently as a regulator of retinal physiology (Du et al., 2017).

There is increasing evidence for impaired vascular EC synthesis and/or bioavailability of H_2_S in diabetes (Szabo, 2012). Lower vascular and/or tissue levels of “H_2_S” have been observed in humans with diabetes (Whiteman et al., 2010a; Suzuki et al., 2017), and in several animal diabetic models, including streptozotocin (STZ)-treated rats (Yusuf et al., 2005; Si et al., 2013; Zhou et al., 2014) and mice (Suzuki et al., 2011) and in db/db (Peake et al., 2013; Sun et al., 2018), ob/ob (Zhao et al., 2017), Akita (Kundu et al., 2013; John et al., 2017), and NOD (Brancaleone et al., 2008) mice. Administration of often high doses of H_2_S *via* sulfide salts at least partially prevented the diabetic phenotype in some of these studies. In the STZ diabetes model specifically, retinal levels of H_2_S were lower in diabetic animals compared with controls (Si et al., 2013), and retinal capillary leakage, VEGF levels, and oxidative stress markers were partially normalized after NaSH treatment (Si et al., 2013). NaSH administration also partially restored mitochondrial function, e.g., increased ATP synthesis and complex II and III activity and prevented mitochondrial oxidant production and mitochondrial swelling, demonstrating a likely mitochondrial target for pharmacological H_2_S (Si et al., 2013). Collectively, these studies strongly suggest that diabetes and diabetic retinopathy (DR) result from “H_2_S deficiency,” which could at least be partially overcome by pharmacological manipulation of mitochondrial H_2_S levels.

It is important to note that these previous studies on H_2_S and diabetes have exclusively relied on the use of sulfide salts to generate H_2_S (e.g., NaSH and Na_2_S). While useful laboratory tools for H_2_S generation, they are not without severe limitations (Whiteman et al., 2011). These include generation of an instant, and unphysiological bolus of H_2_S by pH-dependent dissociation (and rapid decay), whereas endogenous organic enzymatic H_2_S synthesis produces low levels of H_2_S over prolonged periods of time (Li et al., 2008; Whiteman et al., 2010b, 2011; Kabil and Banerjee, 2014).

It is now recognized that mitochondrial oxidative stress may contribute to the pathogenesis of diabetic endothelial dysfunction (Brownlee, 2005) and that in diabetes, the primary target for H_2_S activity appears to be the mitochondria where it is used as an electron source for respiration to reduce oxidative stress (Módis et al., 2014; Szabo et al., 2014). This is supported by data indicating that mitochondrial-targeted slow-release H_2_S donors preserved cellular bioenergetics and increased levels of Nrf2 and peroxisome proliferator-activated receptor-gamma coactivator (PGC-1α), key regulators of mitochondrial antioxidant capacity, bioenergetics, and biogenesis, both *in vitro* and *in vivo* in response to UVA-induced photoaging (Lohakul et al., 2021).

However, H_2_S concentrations (derived from sulfide salts) that are required to reverse mitochondrial dysfunction in the vasculature (e.g., in diabetic rats) are unattainable (Suzuki et al., 2011; Montoya and Pluth, 2016). This is presumably because H_2_S from these tools is generated immediately, locally, and not targeted to mitochondria. With the development of the slow-release cytosolic donor sodium GYY4137 (Li et al., 2008) and the mitochondria-specific sulfide donor AP39 (Le Trionnaire et al., 2014) the limitations of NaSH/Na_2_S have been overcome. The effects on microvascular EC and in the kidney have been previously reported (Ahmad et al., 2016; Gerő et al., 2016), but to the best of our knowledge, no previous studies have reported on the effects of these molecules in DR.

In this current study, we investigated the effects of a single dose (administered by intravitreal injection) of two slow-release H_2_S donor molecules, the cytosolic sodium GYY4137 (Li et al., 2008) and the mitochondria-specific sulfide donor AP39 (Le Trionnaire et al., 2014), on retinal vascular permeability *in vivo* in the Norway Brown rat streptozotocin-induced DR model. In addition, the effects of these two H_2_S generators on *in vitro* EC glycocalyx thickness using bovine retinal endothelial cells (BRECs) were studied. Leucocyte adhesion to BRECs and the effects of the H_2_S modulation were also evaluated in normal and high-glucose conditions.

## Materials and Methods

### H_2_S Donor Synthesis

The mitochondria-targeted H_2_S donor AP39 was synthesized in our laboratories as previously described by our team (Le Trionnaire et al., 2014). Commercially available GYY4137 is a morpholine salt that contains unstated quantities of dichloromethane residual from its initial synthesis and which forms part of the lattice structure (at least 2 dichloromethane:1 GYY4137 molecule) (Alexander et al., 2015), i.e., it is commercially available as xCHCl_2_ or a dichloromethane complex. Dichloromethane is metabolized *in vivo* to carbon monoxide (Ratney et al., 1974; Takano and Miyazaki, 1988). Furthermore, the morpholinium counter ion (1 morpholine:1 GYY4137 molecule) is not biologically inert and has a half-life in rats of ∼90 min (Sohn et al., 1982). LD_50_ values for morpholine in rats and mice, e.g., 100–400 mg/kg for i.p. administration (Kielhorn and Rosner, 1996), are well within the commonly used doses of commercial GYY4137, e.g., 50–300 mg/kg (Lee et al., 2011; Li et al., 2013). In the eye, morpholine and dichloromethane have been reported to cause keratoconjunctivitis, focal/diffuse cataract formation, keratitis, iritis, conjunctival epithelial edema and detachment, and denudation of corneal epithelium (Ballantyne et al., 1976; Brandt and Okamoto, 1988; Toxicology–cosmetic ingredient review, 1989). To avoid these clinical indications and associated toxicity, a cleaner dichloromethane and morpholine-free sodium salt synthesized as previously described was used in this study (Alexander et al., 2015).

### Cell Culture and Treatments

Primary cultures of BRECs were isolated from bovine retinae dissected from eyes of freshly slaughtered cattle, by homogenization and a series of filtration steps as previously described (Chibber et al., 2000). Briefly, excess fat and muscle tissue was removed from the eye globes and the retina were dissected, placed in 20 ml of minimal essential medium (MEM) and homogenized using a hand-held sterile glass homogenizer to dissociate the neural retina. The resulting homogenate was filtered through 80-μm nylon mesh, the material remaining on the mesh was collected, and the trapped microvessels were resuspended in serum free MEM with collagenase-dispase (2 mg/ml) and digested for 90 min at 37°C on a rotator shaker. Following further filtration through a 45-μm polypropylene net filter, the trapped microvessel fragments were vigorously pipetted in MEM with 10% (v/v) pooled human serum, 10% (v/v) tryptose phosphate broth (TPB), L-glutamine 2 mM, penicillin (100 U/ml), and streptomycin (100 μg/ml) and plated in fibronectin-coated tissue culture flasks. After 24 h, the flasks were rinsed once with MEM to remove debris and unattached cells and refilled with fresh growth medium [MEM supplemented with 10% (v/v) pooled human serum, 10% (v/v) tryptose phosphate broth (TPB), L-glutamine 2 mmol/L, and penicillin (100 U/ml) and streptomycin (100 μg/ml)]. From passage 1 onward, the BRECs were cultured on gelatin-coated tissue culture flasks and human serum was replaced with 10% (v/v) horse serum. Pericyte contamination was removed by differential trypsinization. Cultures were >90% pure as assessed by morphology and staining for von Willebrand factor. Preliminary studies confirmed that treatment with 25 mmol/L D-mannitol as osmotic control had no effect on either the glycocalyx or leukocyte adhesion compared with the normal glucose (NG) treatment. U937 cells (human leukemic monocyte lymphoma cell line) were cultured in RPMI-1640 with 10% (v/v) fetal bovine serum, 5.6 mmol/L D-glucose, 25 mmol/L HEPES, and antibiotics. Based on our preliminary results, a final concentration of 500 μmol/L of NaGYY4137 was used in the definitive experiment. NaGYY4137 (500 μmol/L) generates approximately 1 μmol/L or less of H_2_S during the incubation period (Szabo, 2017).

### Glycocalyx Assessment

A cell-based fluorescent assay, based on that described by Singh et al. (2007), was performed to quantify changes within the glycocalyx using fluorescein isothiocyanate-labeled wheat germ agglutinin (WGA-FITC). Bovine retinal endothelial cells were exposed to normal (NG, 5.5 mmol/L) or high glucose (HG, 25 mmol/L) ± GYY4137 (500 μmol/L) in MEM with 0.5% bovine serum albumin (BSA, w/v) for 24 h. Cells were washed 3× with phosphate buffered saline (PBS) and incubated with WGA-FITC [2 μg/ml in HEPES-buffered phenol red-free MEM containing 0.5% BSA (w/v)] for 30 min at 37°C. After washing with PBS (3×), fluorescence intensities were measured (Ex/Em, 485/520 nm) using a fluorescence microplate reader. Cells were lysed and the protein content of each well was measured using the bicinchoninic acid method. Results are presented as fluorescence unit/μg of protein.

### Leukocyte Adhesion Assay

Bovine retinal endothelial cells (BRECs) were cultured to confluency in commercially available μ-slide VI0.4 perfusion slides (Ibidi, München, Germany) coated first with 2% (w/v) bovine gelatin (2 h) and then overnight with bovine fibronectin (50 μg/ml). Cells were treated with NG or HG as above. Calcein acetoxymethyl ester labeled (0.5 μmol/L for 30 min) U937 cells (1 × 10^6^/ml) were then flowed over confluent BREC cultures at a laminar shear stress of 1 dyne/cm^2^ generated by a peristaltic pump. This shear stress was chosen to mimic venular wall shear stresses that favor leucocyte adhesion *in vivo* (Jones et al., 1995; Sundd et al., 2011). After 6 min, slides were washed with PBS for 1 min to remove non-adherent cells, fixed in 4% (w/v) paraformaldehyde, and examined using a Nikon Eclipse TS100 (Nikon UK Limited, Kingston upon Thames, Surrey, United Kingdom) fluorescence microscope to assess the number of adherent U937 cells (10 random fields of view/slide). All experiments were performed on at least three separate occasions. Statistical significance was tested using the Student *t*-test.

### Animal Ethics

Experimental animals were treated in accordance with ARVO Statement for the Use of Animals in Ophthalmic and Vision Research and under a UK Home Office license at the University of Nottingham Biological Services Unit.

### Streptozotocin-Induced Diabetes

A total of 12 male Norway Brown rats (250–300 g, Envigo, United States) were weighed and given a single intraperitoneal (i.p.) injection of STZ (50 mg/kg, Sigma–Aldrich, MO, United States). A total of six control rats were injected with 300 μl of saline i.p. On days 0 and 4 and prior to sacrifice (day 7), blood glucose levels were tested using a sample of blood taken from the tail vein and an Accuchek blood glucose monitor. Rats with blood glucose levels of 15 mmol/L and above were deemed diabetic. Streptozotocin-injected rats that did not become hyperglycemic on day 4 were re-injected with STZ the following morning and subsequently included if deemed diabetic following evaluation for diabetes as outlined above.

### Intravitreal Injections

Rats were anaesthetized with a single 10 mg/ml i.p. injection of Domitor (medetomidine hydrochloride, Pfizer, United Kingdom) and Ketaset (ketamine hydrochloride, Zoetis, NJ, United States). Pupils were dilated with topical applications of 5% (w/v) phenylephrine hydrochloride (Bausch and Lomb) and 0.8% (w/v) tropicamide (Bausch and Lomb), and eyes were coated with Lubrithal (*Dechra)* to prevent dehydration. A 1.5-cm 34-gauge hypodermic needle (Hamilton, NV, United States) attached to a 5-μl syringe (World Precision Instruments, FL, United States) was inserted through pars plana at 3 mm from the limbus into the vitreous of the left eye at a 45° angle. Rats received 5 μl of sterile PBS, 1 μmol/L NaGYY4137 [sodium 4-methoxyphenyl(morpholino)-phosphinodithioate], or 100 nmol/L AP39 [(10-oxo-10-(4-(3-thioxo-3H-1,2-dithiol-5yl)phenoxy)decyl) triphenylphosphonium bromide] on day 0 (prevention arm) or day 6 (treatment arm).

### Fundus Fluorescein Angiography

Angiography was performed as described (Allen et al., 2020). Sodium fluorescein (NaF) dye was prepared by dissolving in sterile PBS to give a final concentration of 100 mg/ml and sterile filtered (0.2 μm). Rats were anesthetized as previously described and fundus images of the retina were captured to check for any ocular abnormalities. Rats received a single 250 μl i.p. injection of sodium fluorescein (100 mg/ml), which was allowed to circulate for ∼60 s before imaging with a Micron IV Retinal Imaging Microscope (Phoenix Research Labs). The green filter was selected, and a 3-min video footage of the retina was recorded at 15 frames per second. This was carried out on days 0 and 7. Development of cataracts with resultant blurring of posterior segment view meant that some eyes (*n* = 2) were excluded from the consecutive fundus fluorescein angiography (FFA).

### Retinal Permeability Model

**FIGURE 1:**
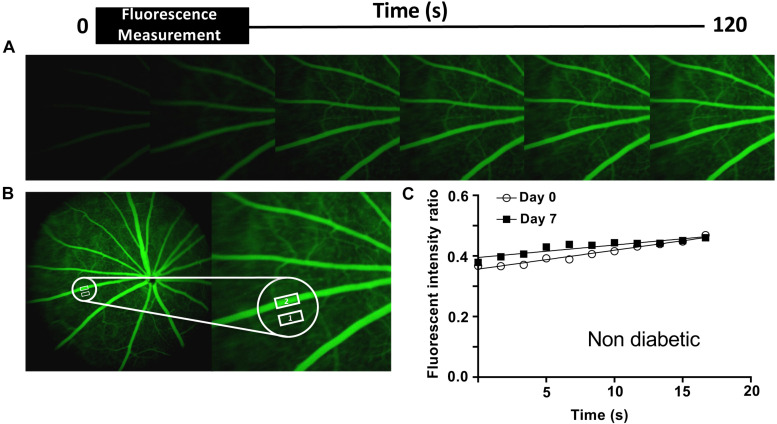
Retinal permeability analysis model. **(A)** Fundus fluorescein angiography was performed in non- and diabetic Norway Brown rats treated ± H_2_S donors, on days 0 and 7. The angiograms shown are representative of a non-diabetic animal. **(B)** The mean intensity of NaF in the retinal tissue (Adamis and Berman, 2008) and a main retinal vessel (Ahmad et al., 2016) were measured every 200 frames for 3 min. **(C)** Apparent permeability (Permeability.Surface Area product, PA) was calculated from the rate of change of extravascular fluorescence (= ΔI_f_/Δt) per unit concentration difference (ΔC) between blood and tissue. P = ΔI_f_/Δt/(ΔC x A). ΔI_f_/Δt ∼ slope. ΔC = difference between intra- and extravascular intensity.

Angiograms were imported into ImageJ software and fluorescence was measured in the interstitium and a major retinal vessel every 200 frames up to 2,400 frames. The ratio of interstitial to vascular fluorescence was adjusted for background level and plotted against time and the slope used to determine an estimate of permeability (Figure 1). Figure 1A shows images taken from rats given NaF and recording started within 1 min of injection. The large vessels of the retina are clearly seen, and the retina becomes brighter over time. To estimate a measure of permeability, the fluorescence intensity in an area with no large vessels was measured. The solute flux (number of fluorescence molecules entering the interstitium per unit time) was calculated from the change in fluorescence intensity in the window outside the major vessel, as a proportion of the fluorescence intensity in the blood vessel in the same frame, to account for any changes in focus, excitation intensity, or noise, plotted against the time in seconds. The permeability surface area product was calculated from the solute flux, the concentration gradient between the intravascular and extravascular compartments (difference in fluorescence intensity), and the area of the extravascular sample measurement according to Fick’s law. In healthy Norway Brown rats, the permeability was the same 7 days after intraocular injection with 2 μl saline (7.47 ± 1.74 × 10^–4^ s^–1^) as it was before injection (6.57 ± 2.13 × 10^–4^ s^–1^). Streptozotocin treatment resulted in a significant increase in blood glucose (29.73 ± 0.66 mmol/L) after 1 week (compared with 7.92 ± 0.37 mmol/L), which was accompanied by a significant increase in solute flux in the retina (Figure 4), which translated to an increase in estimated permeability from 8.19 ± 0.95 × 10^–4^ s^–1^ before STZ to 13.32 ± 1.65 × 10^–4^ s^–1^ after STZ induction (Allen et al., 2020).

### Immunofluorescence

After animal termination and ocular dissection (day 14), retinae were flat-mounted and blocked in 5% (v/v) goat serum, 3% (v/v) Triton X-100, 1% (w/v) BSA, and stained with isolectin-B4 (IB4) (Sigma Aldrich, biotin conjugated) 5 μg/ml and rabbit anti-collagen IV (Abcam) 5 μg/ml overnight at 4°C. Streptavidin conjugated Alexafluor 488 2 μg/ml and donkey anti-rabbit Alexafluor 555 4 μg/ml were used to detect IB4 and collagen IV staining, respectively. Coverslips were mounted with Fluoroshield with DAPI. Images were obtained using a Leica TCS SPE confocal microscope, and all settings were maintained between images.

### Statistics

All statistics and graphs were produced in GraphPad Prism 6 and statistical tests are shown in figure legends. Significant differences are indicated on graphs as asterisks, where: ^∗^*p* ≤ 0.05; ^∗∗^*p* ≤ 0.01; ^∗∗∗^*p* ≤ 0.001.

## Results

### Glycocalyx Integrity

Two hydrogen sulfide generating molecules were investigated, NaGYY4137 and AP39. The former is a slow-releasing hydrogen sulfide donor (Alexander et al., 2015) that does not target any specific part of the cell, whereas AP39 is a mitochondrial targeted donor (Le Trionnaire et al., 2014).

**FIGURE 2:**
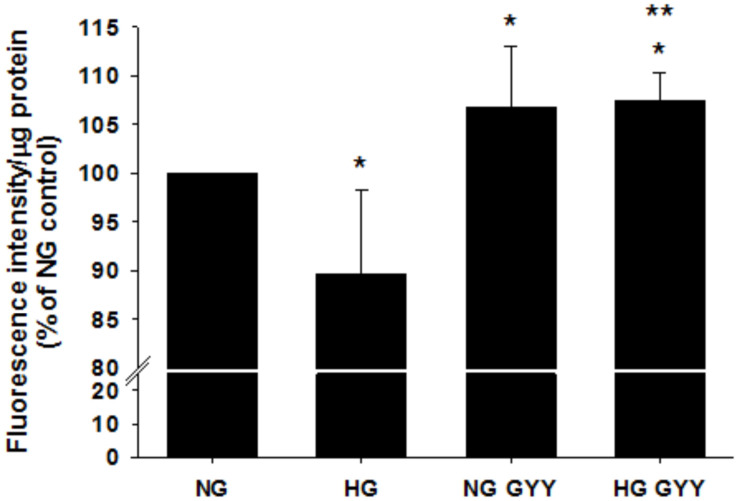
Administration of the slow-release H_2_S donor NaGYY4137 completely reversed the loss of retinal EC glycocalyx integrity induced by hyperglycemia. Retinal ECs were incubated with normal (5.5 mmol/L, NG) or high glucose (25 mmol/L, HG) ± GYY4137 (500 μmol/L) for 24 h before assessment of glycocalyx integrity with fluorescently labeled lectin (WGA-FITC). Data are mean ± SD, presented as fluorescence intensity/μg protein. *p ≤ 0.01 vs. control, **p ≤ 0.01 vs. HG, *n* = 12–30. One-way ANOVA.

HG significantly reduced BREC glycocalyx *in vitro* (assessed by FITC-WGA staining) to 89.67% ± 6.6% compared with NG control conditions (100%), *p* ≤ 0.001, *n* = 12–30 (Figure 2). Simultaneous treatment with the slow-release H_2_S donor NaGYY4137 completely reversed this loss and actually restored the glycocalyx to higher levels than observed in the NG control (107.49 ± 8.13% vs. 100%, *p* ≤ 0.001, *n* = 12–30). Interestingly, cells incubated in NG conditions in the presence of NaGYY4137 also had significantly higher glycocalyx staining than control NG-treated cells (106.7 ± 6.35 vs. 100%, *p* ≤ 0.001, *n* = 12–30).

**FIGURE 3:**
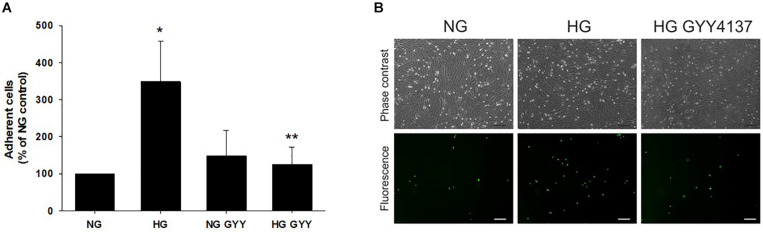
Administration of the slow-release H_2_S donor NaGYY4137 completely attenuated the increased leukocyte adhesion to retinal ECs induced by hyperglycemia. Retinal ECs were incubated with normal (5.5 mmol/L, NG) or high glucose (25 mmol/L, HG) ± NaGYY4137 (500 μmol/L) for 24 h before examination of leukocyte adhesion under flow (1 dyne/cm^2^). **(A)** Number of adherent U937 cells. Data are mean ± SD. *p ≤ 0.001 vs. control, **p ≤ 0.001 vs. HG, *n* = 4. One-way ANOVA. **(B)** Representative bright field, phase contrast (upper panel) and corresponding fluorescence images (lower panel) of adherent U937 cells stained with calcein acetoxymethyl ester adhering to a BREC monolayer. Scale bar, 75 μm; original magnification, ×100.

Since the integrity of the glycocalyx may alter endothelial cell adhesion molecule exposure and thus influence leukocyte/endothelial binding, adhesion of U937 leukocytes to a BREC monolayer was examined under the same experimental conditions. HG significantly increased leukocyte adhesion to 348 ± 110% vs. NG control (100%), *p* ≤ 0.01, *n* = 4 (Figure 3). This increase was fully attenuated to control levels by incubation with NaGYY4137 (HG + NaGYY4137; 124 ± 47% vs. HG; 348 ± 110%, *p* ≤ 0.01, *n* = 4). NaGYY4137 had no significant effect on leukocyte binding in NG conditions.

**FIGURE 4:**
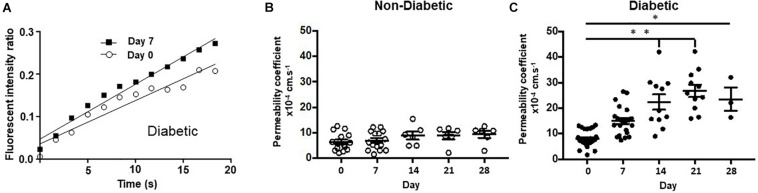
Chemically induced diabetes significantly increased retinal vascular permeability in Norway Brown rats compared to non-diabetic controls. **(A)** Fundus fluorescein angiography measurements from a single rat carried out on day 0 (before STZ) and day 7 (1 week of STZ treatment). **(B)** Permeability was measured from control (blood glucose 7.99 ± 0.21 mmol/L), and **(C)** diabetic (blood glucose 29.75 ± 1.63 mmol/L) Norway Brown rats on days 0, 7, 14, 21, and 28. Apparent permeability was calculated as described in the methodology text and depicted in Figure 1. Data including mean ± SEM. *p ≤ 0.001 vs. day 0, **p ≤ 0.0001 vs. diabetic day 0. One-way ANOVA.

### Retinal Permeability

To determine the effect of hydrogen sulfide donors on retinal permeability, fluorescein fundus angiography was carried out before and after induction of diabetes with STZ. NaF was injected intraperitoneally into Norway Brown rats and the fluorescence intensity inside a large vessel and in the tissue outside a large vessel was determined over time by video-microscopy.

**FIGURE 5:**
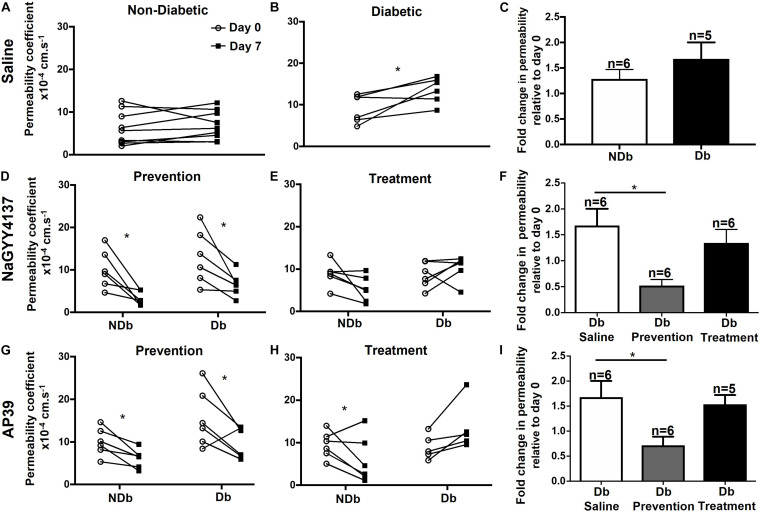
Ocular administration of the slow-release H_2_S donors reduced retinal permeability in chemically induced diabetic rats. Type I diabetes was successfully induced in rats following a single dose of STZ and animals were maintained for 1 week without insulin supplementation. Retinal vascular leak was measured on day 0 and day 7 in non- and diabetic animals following treatment. No observed increases in retinal permeability were measured on day 7 in non-diabetic animals treated with saline **(A,C)**. In diabetic animals treated with saline a significant (*p ≤ 0.05) increase in retinal permeability was measured on day 7 vs. day 0 **(B,C)**. NaGYY4137 and AP39 significantly (*p < 0.05, paired *t*-test) reduced retinal permeability in non- and diabetic rats **(D,G,F)** on day 7 when given as a single intraocular preventative dose, on day 0. In addition, NaGYY4137 and AP39 reduced retinal permeability in non-diabetic rats **(E,F,H,I)** when administered therapeutically (day 6). In diabetic rats, NaGYY4137 and AP39 stabilized retinal permeability on day 7 in rats with pre-existing diabetes **(E,F,H,I)**.

Retinal vascular permeability was significantly increased in the diabetic Norway Brown rats (23.51 ± 4.59 × 10^–4^ s^–1^, *n* = 3) compared with normal control rats (9.38 ± 1.40 × 10^–4^ s^–1^, *n* = 6, Figure 4) on day 28 (*p* ≤ 0.001) on day 28 and all other time points. In the H_2_S donor treatment study no changes in retinal permeability were measured on day 7 compared to baseline in non-diabetic animals treated with vehicle control (saline) (Figure 5A). However, a significant (*p* ≤ 0.05) increase in retinal permeability was measured in non-insulin treated diabetic animals on day 7 (12.16 ± 0.98 × 10^−4^ s^−1^) compared to baseline (9.23 ± 1.54 × 10^−4^ s^−1^) (Figures 5B,C). The slow-release hydrogen sulfide donor NaGYY4137 significantly reduced retinal vascular permeability in control, non-diabetic rats after 7 days (3.24 ± 0.66 × 10^–4^ s^–1^, *p* ≤ 0.05, *n* = 6) vs. day 0 (10.12 ± 1.85 × 10^–4^ s^–1^) when administered as a single preventative intraocular dose pre-diabetes (Figure 5D). Similarly, NaGYY4137 given in diabetic rats significantly reduced retinal permeability on day 7 (6.62 ± 1.16 × 10^–4^ s^–1^, *p* ≤ 0.05, *n* = 6) vs. day 0 (13.08 ± 2.61 × 10^–4^ s^–1^) when given as a preventative treatment (Figure 5D). When NaGYY4137 was administered therapeutically, retinal permeability was reduced in control, non-diabetic animals on day 7 (5.31 ± 1.24 × 10^–4^ s^–1^, *n* = 6) vs. day 0 (8.90 ± 1.19 × 10^–4^ s^–1^, *n* = 6). More importantly, in animals with pre-existing diabetes, retinal permeability stabilized on day 7 (10.27 ± 1.21 × 10^–4^ s^–1^, *n* = 6) compared to day 0 (8.65 ± 1.23 × 10^–4^ s^–1^, *n* = 6) (Figure 5E). Administering NaGYY4137 treatment prior to the onset of diabetes gave a 68% benefit in reducing retinal permeability compared with administration to animals with pre-existing diabetes (Figure 5F).

Similarly, the mitochondrial-targeted hydrogen sulfide donor AP39 significantly reduced retinal permeability in control, non-diabetic (6.14 ± 0.90 × 10^–4^ s^–1^, *p* ≤ 0.05, *n* = 6) and diabetic rats (9.84 ± 1.48 × 10^–4^ s^–1^, *p* ≤ 0.05, *n* = 6) compared with day 0 (10.01 ± 1.34 × 10^–4^ s^–1^ and 15.52 ± 2.75 × 10^–4^ s^–1^, respectively) when administered as a single preventative dose (Figure 5G). In addition, AP39 reduced retinal permeability after a week (5.93 ± 2.25 × 10^–4^ s^–1^, *n* = 6) compared with baseline (9.49 ± 1.28 × 10^–4^ s^–1^, *p* ≤ 0.05, *n* = 6) in control animals when administered therapeutically. However, in diabetic animals, AP39, when administered therapeutically, stabilized retinal permeability (13.63 ± 2.56 × 10^–4^ s^–1^, *n* = 5) on day 7 versus baseline (8.99 ± 1.31 × 10^–4^ s^–1^, *n* = 5) (Figure 5H). Similar to NaGYY4137, administering AP39 treatment prior to the onset of diabetes gave an 18% benefit in reducing retinal permeability when compared with animals with pre-existing diabetes (Figure 5I). Overall, both H_2_S donors were more effective in reducing retinal permeability when given to animals prior to the onset of diabetes. In addition, NaGYY4137 showed greater efficacy than a 10-fold lower dose of the mitochondrial targeted AP39 compound.

### Acellular Capillary Formation

**FIGURE 6:**
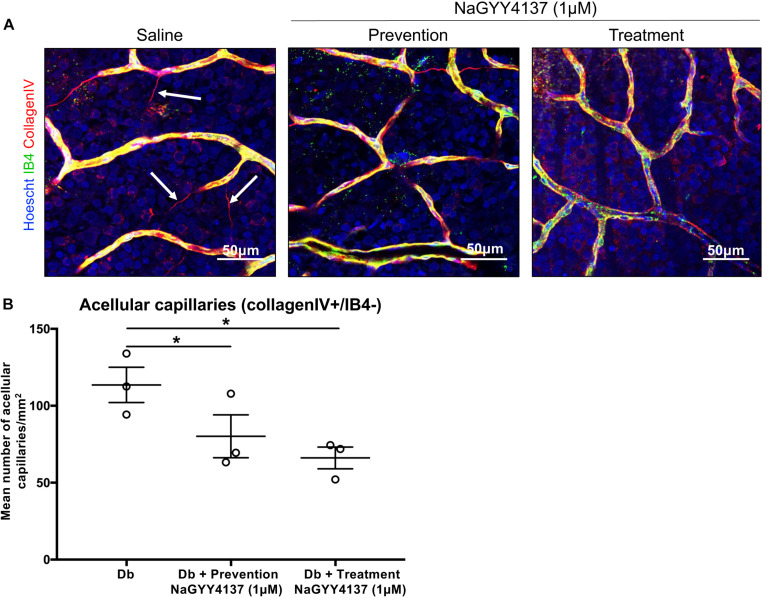
NaGYY4137 protected against diabetes-induced increase in acellular capillaries. Norway Brown rats were given a single dose of streptozotocin (50 mg/kg) to induce Type I diabetes. Rats were treated with an intraocular injection of either saline or 1 μmol/L H_2_S donor NaGYY4137 at day 0 (prevention) or day 6 (treatment). **(A)** Rats were enucleated at day 7 and whole retinae were mounted and stained with Hoescht, IB4, and collagen IV. The tissue was imaged using 20 × lens. The number of capillaries positively stained for collagen IV but lacking IB4 (white arrows) were counted in three areas of the retina. **(B)** Acellular capillaries are expressed per mm^2^. Error bars represent standard errors of the mean. Statistical analysis was performed using a one-way analysis of variance corrected for false discovery rate. *p ≤ 0.05.

One of the contributing factors to the pathogenesis of DR is the presence of acellular capillaries within the retina. These vessels maintain a basement membrane but have lost the supporting pericytes and EC cells and thus have no blood flow, promoting retinal ischemia. In order to assess the effects of NaGYY4137 on acellular capillary formation, retinae were collected at the end of the retinal permeability study and stained for isolectin B4, an EC marker, and collagen IV, a major constituent of the basement membrane. NaGYY4137 was found to reduce the number of acellular capillaries (collagen IV + /IB4-) in the diabetic retinae in both the prevention and treatment group to 80.2 ± 13.9 mm^–2^ and 66.1 ± 7.1 mm^–2^, respectively, compared with 113.6 ± 11.7 mm^–2^ in the diabetic retinae (*p* ≤ 0.05, *n* = 3, Figure 6). Despite the increased effectiveness of NaGYY4137 in reducing retinal permeability when administered prior to diabetes onset, the time of administration appeared to have no difference in acellular capillary formation.

## Discussion

The data presented here indicate, for the first time, that (a) high glucose-driven glycocalyx loss, (b) high glucose-enhanced leukocyte adhesion to the endothelium, and (c) diabetes-associated increases in retinal permeability can all be reversed by administration of a slow-release H_2_S donor.

Hyperglycemia-related low-grade persistent inflammation is thought to contribute to the pathology of early DR (Adamis and Berman, 2008). Patients with DR have elevated ocular levels of inflammatory mediators and the diabetic retina displays characteristic features of inflammation including increased vascular permeability and leukocyte adhesion (Adamis and Berman, 2008). Since a healthy glycocalyx is critical to maintenance of both the anti-inflammatory and permeability characteristics of a healthy endothelium, our data highlight the potential of H_2_S donors as new therapeutic tools for the treatment of DR. Further investigations into optimal dosing and comparing other H_2_S delivery molecules are needed to further explore this potential.

The available literature describing other research in this area is scarce. However, glycocalyx loss and enhanced leukocyte adhesion have been reported in a rat model of diabetes (Kumase et al., 2010). Additionally, a clear link between *in vivo* retinal glycocalyx integrity and vascular permeability has been reported by Leskova et al. (2019), who demonstrated, in mice, that enzymatic degradation of the glycocalyx is associated with enhanced retinal permeability. These data support the hypothesis that maintaining glycocalyx integrity could be a viable therapeutic strategy for DR. EC glycocalyx thickness depends on the rate of shedding and circulatory levels of glycosaminoglycans (GAG)-degrading enzyme levels (Ikegami-Kawai et al., 2003; Maxhimer et al., 2005). The previously reported association of glycocalyx reduction with increased vascular permeability and leucocyte and platelet adhesion and subsequent reversal by glycocalyx restoration in experimental animals through increased GAG synthesis (Henry and Duling, 1999; Constantinescu et al., 2003) corroborate our findings. Furthermore, Ciszewicz et al. (2009), have shown that sulodexide attenuates hyperglycemia-associated EC permeability and inflammation. Similarly, Broekhuizen et al. (2010), demonstrated that sulodexide increased vascular EC glycocalyx and reversed the increased vascular permeability in type 2 diabetes. Additionally, Niu et al. (2019), using the STZ-induced diabetic rat model used here, showed that endomucin overexpression restored the diabetes-associated loss of retinal endothelial glycocalyx and that this restoration was associated with decreased leukocyte–endothelial adhesion and a reduction in vessel leakage in rats with DR.

The effects of H_2_S on the glycocalyx are still unknown. Although early studies showed potential antioxidant effects of H_2_S, more recent work has shown that the reaction rate between H_2_S and oxidant species, e.g., peroxide and peroxynitrite, may be too slow for oxidant scavenging activity alone to be primarily responsible for glycocalyx- and vaso-protection, especially given the low *in vivo* concentrations of H_2_S, which we have modeled using NaGYY4317 (Carballal et al., 2011). This strongly suggests that H_2_S is acting *via* other mechanisms to maintain the glycocalyx during hyperglycemic damage, e.g., enhancing synthesis of glycocalyx components or preventing hyperglycemia-induced glycocalyx degradation. For instance, the glycocalyx component syndecan-1 is cleaved by matrix metalloproteinase-9 (MMP-9) and both activity and expression of the protease are known to be reduced by H_2_S administration (Du et al., 2017). It has also been reported that MMP-7 can cleave chondroitin sulfate (Lipowsky, 2011) and syndecans 1 and 4 (Manon-Jensen et al., 2013) and our unpublished data indicate that NaGYY4137 inhibits MMP-7 in *in vitro* assays at concentrations similar to those used in the studies described here. Interestingly, our additional preliminary data indicating that NaGYY4137 reduces shedding of hyaluronic acid from cultured ECs suggest another potential action of H_2_S since the significant reduction in EC glycocalyx observed in acute hyperglycemia (Nieuwdorp et al., 2006b) and in types 1 and type 2 diabetes (Nieuwdorp et al., 2006a; Broekhuizen et al., 2010) is thought to be related to increased hyaluronidase catabolism (Nieuwdorp et al., 2006a).

It is interesting to note that NaGYY4137 treatment in NG enhanced glycocalyx staining above the levels observed in NG alone (Figure 2), suggesting that H_2_S supplementation alone increased glycocalyx density. As mentioned previously, the glycocalyx is a dynamic structure and thus this observation could potentially be explained by the ability of H_2_S to modify glycocalyx-degrading enzymes to reduce their activity. Interestingly, H_2_S is the only molecule known to induce protein *S*-persulfidation (*S*-sulfhydration), a recently identified and unique post-translational protein modification. It is possible that the activity of glycocalyx-degrading enzymes such as heparinase might be altered by persulfidation of the cysteine residues of its active site. Additionally, cysteine residues in proteins have the potential to be redox modified by H_2_S in a non-S-persulfidation manner and several potential glycocalyx-degrading enzymes, including the proteinases MMP-1 (Lohakul et al., 2021), MMP-2, MMP-7, and MMP-9, have a cysteine in their active site. Thus, as has been shown with other zinc proteases including angiotensin-converting enzyme (Laggner et al., 2007) and TNF-α-converting enzyme (Li et al., 2013), H_2_S has the potential to modulate the glycocalyx-degrading enzyme activity of the proteinases, although further work is needed to confirm this hypothesis.

The effectiveness of our mitochondrial targeted H_2_S donor in decreasing retinal leakage *in vivo* raises the possibility of mitochondrial involvement in the observed effects. Modis et al. reported that endogenous mitochondrial H_2_S production was governed by MST (Módis et al., 2013). Additionally, Si et al. (2013) reported that DR was associated with significant decreases in retinal H_2_S levels and expression of the H_2_S synthesizing enzymes CSE, CBS, and 3MST (i.e., DR resulted in retinal H_2_S deficiency) and also with increased mitochondrial permeability and respiration (Si et al., 2013), strongly suggesting extensive mitochondrial dysfunction and H_2_S deficiency in the retina in DR. Vascular mitochondrial impairment induced by diabetes has previously been shown to be inhibited/reversed by sulfide administration albeit at high doses/concentrations (e.g., 300 μmol/L) (Módis et al., 2013; Coletta et al., 2015), suggesting that pharmacological sulfide could overcome impaired retinal bioenergetics in DR. Although NaSH administration has previously been shown to reduce retinal vascular abnormalities associated with DR and “correct” mitochondrial dysfunction (Si et al., 2013), this earlier study is intriguing for several reasons. Firstly, the NaSH was given as a daily intraperitoneal injection (over 14 weeks) at a dose equivalent to 280 mmol/kg. This was an exceptionally high and surprisingly non-lethal dose (although toxicity was not examined) given that 14 μmol/kg i.p. induces systemic inflammation and vascular collapse (Li et al., 2005) and the LD_50_ for NaSH is 52.6 μmol/kg for the same route of administration (Strickland et al., 2003). Possible toxicological constraints aside, the half-life of bolus sulfide in blood is less than a minute as it is rapidly metabolized/removed (Wang, 2002, 2012), so the precise mechanisms by which a bolus of H_2_S (from NaSH), administered intraperitoneally survives intact to penetrate the blood–brain barrier and selectively increase sulfide levels in the retina *via* this route remain elusive. Given that plasma H_2_S levels have been measured at nanomolar to low micromolar concentrations, our approach to prevent/reverse DR-induced retinal vascular leakage using slow-release H_2_S donors (Wei et al., 2014; John et al., 2017; Qiu et al., 2018) or H_2_S delivery molecules that selectively target mitochondria (Le Trionnaire et al., 2014; Szczesny et al., 2014; Ikeda et al., 2015; Ahmad et al., 2016; Karwi et al., 2017) would overcome these concerns.

## Conclusion

The data presented here together with previous *in vivo* data (Whiteman et al., 2010a) suggest that, in health, H_2_S is vasculoprotective and that microvascular dysfunction in diabetes, including DR, is associated with reduced circulating and retinal H_2_S levels. Thus, diabetes may be a condition of H_2_S deficiency and H_2_S supplementation using slow-release and/or mitochondrial-targeted H_2_S delivery molecules may represent a novel, cost-effective alternative therapy for DR.

## Data Availability Statement

The original contributions presented in the study are included in the article/supplementary material. The raw data supporting the conclusions of this article will be made available by the authors, without undue reservation.

## Ethics Statement

The animal study was reviewed and approved by University of Nottingham Biological Services Unit.

## Author Contributions

JW, DB, and MWh conceived and planned the experiments. CA, JW, DB, and MWh took the lead in writing the manuscript. CA, KW, and NM carried out the experiments. RT and MWo synthesized the slow-release hydrogen sulfide donors. AB and WA contributed to the interpretation of the results. All authors provided critical feedback and helped shape the research, analysis, and manuscript.

## Conflict of Interest

MWh, RT, and MWo, and the University of Exeter have intellectual property (patent filings) related to hydrogen sulfide delivery molecules and their therapeutic use. MWh was a consultant to MitoRx Therapeutics (Oxford). The remaining authors declare that the research was conducted in the absence of any commercial or financial relationships that could be construed as a potential conflict of interest.

## Publisher’s Note

All claims expressed in this article are solely those of the authors and do not necessarily represent those of their affiliated organizations, or those of the publisher, the editors and the reviewers. Any product that may be evaluated in this article, or claim that may be made by its manufacturer, is not guaranteed or endorsed by the publisher.
